# Anatomical findings in patients with chronic rhinosinusitis without nasal polyps requiring revision surgery

**DOI:** 10.1016/j.bjorl.2023.101287

**Published:** 2023-07-03

**Authors:** Anna Sophie Englhard, Georg Johannes Ledderose

**Affiliations:** a Klinikum der Universität München, Department of Otorhinolaryngology ‒ Head and Neck Surgery, Marchioninistr, Munich, Germany; b ENT-Center Dr. Lübbers & Kollegen, Weilheim, Germany

**Keywords:** Revision sinus surgery, Functional endoscopic sinus surgery, Chronic rhinosinusitis, FESS, CRSsNP

## Abstract

**Objectives:**

For the treatment of chronic rhinosinusitis functional endoscopic sinus surgery is a well-established therapy with high initial success rates. However, a significant proportion of patients have persistent disease requiring revision surgery. To date, studies including data of large patient collectives are missing. In this study, we aimed to identify anatomic factors increasing the need for revision surgery in a large patient collective with chronic rhinosinusitis without nasal polyps.

**Methods:**

Data were collected retrospectively on patients with recurrent or persistent chronic rhinosinusitis without nasal polyps requiring revision surgery. The patients’ symptomatology, endoscopic and radiographic findings were analyzed. Preoperatively, patients were evaluated with endoscopic examination of the nose and paranasal sinuses. In all individuals computed tomography of the sinuses was performed. Images were evaluated according to the Lund-Mackay system. Information was also collected intraoperatively.

**Results:**

253 patients were included. The most common anatomic factor was incomplete anterior ethmoidectomy (51%), followed by residual uncinated process (37%), middle turbinate lateralization (25%), incomplete posterior ethmoidectomy (20%), frontal recess scarring (19%), and middle meatal stenosis (9%). Other factors such as persistent sphenoid pathology was less frequent.

**Conclusion:**

Iatrogenic causes with inadequate resection of obstructing structures seem to be a principal risk factor for recurrent chronic rhinosinusitis and the need for revision sinus surgery. Meticulous attention in the area of the ostiomeatal complex during surgery with ventilation of obstructed anatomy as well as avoidance of scarring and turbinate destabilization may reduce the failure rate after primary endoscopic sinus surgery.

**Level of evidence:**

2b.

## Highlights

• Data from a large patient collective identify risk factors for recurrent sinusitis.
• Inadequate surgery determines to a large extent the need for revision sinus surgery.
• Special attention in the area of the ostiomeatal complex may reduce the failure rate.

## Introduction

Over the past three decades, Functional Endoscopic Sinus Surgery (FESS) has become a well-established therapy for the treatment of Chronic Rhinosinusitis (CRS).^1^ Published success rates vary from 76% to 98%.^2^, ^3^ However, a significant proportion of patients have persistent disease despite surgical treatment, with as many as 10%–17% requiring revision surgery.^4^, ^5^ Nasal polyposis is a common cause for revision surgery; however, a significant number of patients with Chronic Rhinosinusitis without Nasal Polys (CRSsNP) suffer from persistent CRS despite surgical treatment. Multiple factors are implicated in the failure of FESS, including environmental, host and iatrogenic etiologies.^6^

Revision FESS represents a challenge to all who practice sinus surgery. Compared to primary surgery, success rates of revision sinus surgery are reduced to about 70%.^7^ Moreover, revision surgery has increased complication rates. One the most important considerations in revision sinus surgery is the identification of the anatomy that is contributing to the disease process and the patient’s symptoms. The diagnostic evaluation should include meticulous nasal endoscopy and Computed Tomography (CT) in order to assess the anatomical variation, areas of scarring and remnant bony partitions.^6^ Several authors investigated the etiology of the primary FESS failure.^8^, ^9^, ^10^, ^11^, ^12^ However, most studies included only small patient cohorts. Recent reports examining anatomical and radiological characteristics that may contribute to recurrent disease concluded that trials with larger numbers of patients are necessary.^13^, ^14^

In the presented study, we retrospectively analyzed the data of a comparatively large number of patients with CRS who underwent revision sinus surgery. In particular, we focused on endoscopic and radiological findings in order to identify common anatomical factors that may predispose persistent or recurrent CRS. As nasal polyposis is a common cause of persistent disease despite well-executed surgical treatment, we included only patients with CRSsNP.

## Methods

In the presented study, we analyzed anonymized data retrospectively. In accordance with the specifications of the university’s ethics committee, in this case the informational right of self-determination was not affected, as personal data was not investigated. For this reason, there was no obligation for a formal consultation. An official ethics’ committee statement was not necessary.

Patients who underwent revision FESS for CRSsNP at the department of otorhinolaryngology, head, and neck surgery of the Ludwigs Maximilians University, Munich, Germany, and at the ENT-Clinic Munich Bogenhausen, Dr. Gaertner GmbH, Munich, Germany, were included in this retrospective study. A total of 253 cases presenting with persistent or recurrent CRSsNP and the need for revision surgery were identified. Patients with a history of benign or malignant tumors were excluded. As nasal polyposis is a common underlying etiology of persistent disease and we aimed to concentrate on the anatomic factors causing failure of FESS, patients with CRSwNP were also excluded.

The patients’ symptomatology, endoscopic and radiographic findings were analyzed in this study. In all individuals a pre-, and intraoperative endoscopic evaluation of the nose and paranasal sinuses was performed. Each side was categorized separately. All patients had preoperative high-resolution CT scans. Images were evaluated according to the Lund-Mackay system (0, 1, 2 points given to each sinus separately for no, partial or complete opacification, respectively; and 0 or 2 points given to a non-obstructed or obstructed ostiomeatal complex).^15^ Each side was categorized separately. Moreover, anatomical abnormalities that were recognized on the preoperative CT scans and the intraoperative endoscopic findings were recorded. Anatomical variants of the frontal sinus were categorized according to the International Classification of the radiological Complexity (ICC).^16^

## Results

### Study population

253 individuals presenting with recurrent CRSsNP and the need for revision FESS were included in this study. 401 sides were operated. 39% of the patients were female. The mean age was 51 years (8–85 years).

### Symptoms

The most common symptoms patients complained about were nasal congestion (46%) and pain (42%) (Table 1). 38% of all individuals noted a sensation of pressure over the paranasal sinus. 33% reported recurrent episodes of sinusitis. 18% suffered from hyposmia and 18% from nasal discharge.

**Table 1:** Preoperative symptoms.

Symptoms	Frequency (%)
Sensation of pressure	38
Pain	42
Nasal discharge	18
Nasal congestion	46
Hyposmia	18
Recurrent sinusitis	33
Other	27

### Previous surgeries

62% of all patients had a history of one previous FESS. 25% of all cases had been operated twice and 13% three or more than three times.

### Preoperative evaluation

**Figure 1:**
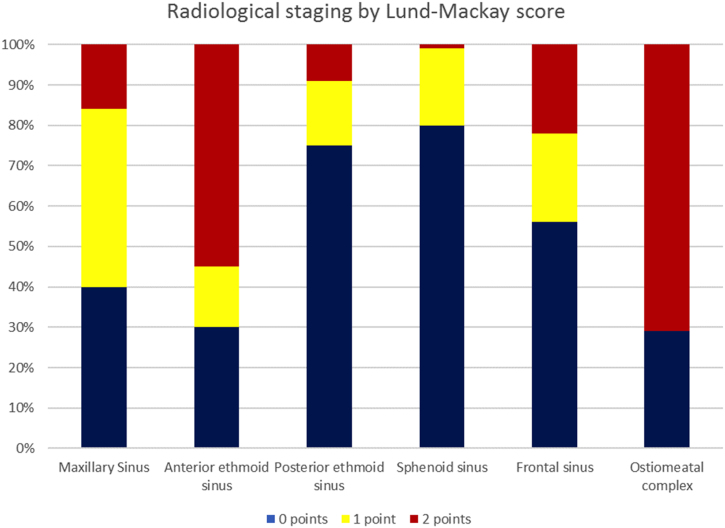
Preoperative radiological staging by Lund-Mackay (506 sides). 0, 1, 2 points are given to each sinus separately for no, partial, or complete opacification, respectively; and 0 or 2 points given to a non-obstructed or obstructed osteo-meatal complex).

All patients underwent preoperatively routine diagnostics including endoscopic examination and CT scans. High-resolution CT scans were evaluated according to the Lund-Mackay system (Fig. 1). Each side was categorized separately. The most common radiological finding was opacification of the anterior ethmoid sinus (partial 15%, complete 55%), followed by opacification of the maxillary sinus (partial 44%, complete 16%). In 44% the frontal sinus was opacified (partial 22% and complete 22%) and 25% the posterior ethmoid sinus (partial 16% and complete 9%). Opacification of the sphenoid sinus was seen in 20% of all cases (partial 19% and complete 1%).

### Anatomical abnormalities

Several anatomical abnormalities were noted on the preoperative CT scans and endoscopically during revision FESS (Table 2, Table 3). The most common finding was incomplete anterior ethmoidectomy (51%). In 37% a residual uncinated process was found. 33% of all cases had recirculation phenomena. Incomplete posterior ethmoidectomy was diagnosed in 20% of patients. Middle turbinate lateralization was observed in 25%. Middle Meatal Antrostomy (MMA) stenosis was seen in 9%. 19% of all patients had frontal recess scarring. A concha bullosa was found in 8%. 4% of all individuals were diagnosed with a persistent infraorbital cell. A persistent sphenoid pathology and ostium stenosis was diagnosed in 7%. A persistent sphenoethmoidal cell was seen in 1% of all cases.

**Table 2:** Anatomical abnormalities noted on the preoperative CT scans and with intraoperative endoscopy of 253 patients.

Anatomical findings	Number	%
Middle turbinate lateralization	62	25
Concha bullosa	19	8
Residual uncinated process	94	37
MMA stenosis	22	9
Recirculation phenomena	84	33
Frontal recess scaring	47	19
Incomplete anterior ethmoidectomy	129	51
Incomplete posterior ethmoidectomy	51	20
Persistent infraorbital cell	9	4
Persistent sphenoethmoidal cell	2	1
Persistent sphenoid pathology and ostium stenosis	18	7

MMA, Middle Meatus Antrostomy.

**Table 3:** Classification of frontal sinus anatomy in 401 sides (253 patients) (ICC classification, Wormald et al.).

Classification of frontal sinus anatomy	Number	%
ANC, SAC, SBC	66	16
SAFC	24	6
FSC	4	1
SBFC	16	4
SOEC	4	1

ANC, Agger Nasi Cell; SAC, Supra Agger Cell; SAFC, Supra Agger Frontal Cell; FSC, Frontal Septal Cell; SBC, Supra Bulla Cell; SBFC, Supra Bulla Frontal Cell; SOEC, Supra-Orbital Ethmoid Cell.

With regard to the frontal sinus anatomy, findings were categorized according to the ICC classification system (Table 3). Each side was categorized separately. The most common abnormalities were agger nasi cells, supra agger cells or supra bulla cells (16%). In 6% of all cases supra agger frontal cells was found. Supra bulla frontal cells were seen in 4%. Both, frontal septal cells, and supra-orbital ethmoid cells were rare and observed in only 1% of all cases.

## Discussion

Failure of primary endoscopic sinus surgery to alleviate sinonasal symptoms and eliminate mucosal disease can be caused by a number of etiologies, systemic and local. Patients with underlying systemic illness such as cystic fibrosis, Samter’s triad, primary ciliary dyskinesia and immunodeficiencies may have recurrent disease despite well-executed primary surgery.^6^, ^17^, ^18^ For this reason, only patients with CRSsNP were included in the presented study.

Several authors investigated the etiology of primary FESS failure. However, most reports included only small patient cohorts, ranging from 24 to 80 patients.^8^, ^9^, ^10^, ^12^, ^13^, ^14^, ^19^ A recent investigation examining anatomical characteristics that may contribute to recurrent disease concluded that trials with larger numbers of patients are necessary.^13^ In presented study, we analyzed the data of the comparatively large number of 253 patients.

Patients requiring revision FESS mostly present with considerable symptoms, including pain, nasal congestion and hyposmia. Accordingly, endoscopic, and radiologic findings are pronounced. The use of standardized scores is of great value for the objectification of these findings and to enable a comparison with other investigations. There are several systems available to stage disease severity in CRS. The radiologic staging by Lund–Mackay^15^ is most commonly used and correlates well with other markers of disease severity, the nature of surgery offered, the number of complications, and revision rates.^20^ The mean normal score in patients who undergo imaging for other than sinonasal symptoms has been reported to be 4.3.^21^ It has therefore been proposed that patients submitted to endoscopic sinus surgery for CRS should have a minimum score of 4. In the current investigation most patients had an obstructed ostiomeatal complex and high scores in the Lund-Mackay system. This is comparable to the results of other studies describing radiologic findings of patients undergoing revision FESS.^8^, ^13^

In 1995, Lund and Kennedy, heading the Staging and Therapy Group for Chronic Rhinosinusitis, proposed the Lund–Kennedy (LK) endoscopic scoring system based on the degree of scarring, crusting, edema, polyps, and discharge.^8^

To date, LK system remains the most frequently utilized and referenced endoscopic scoring system in rhinology outcomes.

The main purpose of this study was to identify common anatomical – and therefore surgical – factors that may predispose to recurrent CRS. Nasal polyposis is known to be an important underlying factor for primary surgical failure and may reduce the surgical success rate.^5^ For this reason, we excluded patients presenting with CRSwNP.

Several studies report inadequate resection of obstructing structures to be a principal risk factor for recurrent CRS.^8^, ^10^, ^13^, ^19^ This is in line with our results of incomplete anterior in 51% and incomplete posterior ethmoidectomy in 20% of all cases. Most other investigations found similar rates of incomplete ethmoidectomy ranging from 31% to 72%.^10^, ^11^, ^13^, ^19^ Khalil et al.^8^ examined the radiological findings in 63 patients undergoing revision sinus surgery. They observed residual anterior and posterior ethmoid cells in 97% and 92% of patients, respectively. The authors speculate that these high rates of incomplete ethmoidectomy might be due to more conservative FESS techniques practiced in the U.K. in comparison to the U.S.

A residual uncinated process was seen in 37% of our patients. This is comparable to the results of other investigations.^10^ Baban et al.^13^ examined the radiological and anatomical findings of 24 patients undergoing revision FESS. They found a residual uncinated process in 52% of their patients. Gore et al.^19^ and Khalil et al.^8^ reported rates of 64% of patients (46% of sides) and 60% (57% of sides), respectively. Incomplete removal of the uncinate process may obscure the position of the natural maxillary sinus ostium. In this situation, the surgical MMA may be created without a communication to the natural ostium. The result may be a recirculation phenomenon, which was observed in 33% of our patients. This may lead to nasal discharge, crusts and opacified sinuses – depicted in the high scores in the Lund-Mackay system. Parsons et al.^22^ described the “missed ostium sequence” as the most important cause of failure of primary FESS. Other studies reported slightly smaller rates of recirculation phenomena of 4%–15%.^10^, ^11^

Previous investigations have identified a lateralized middle turbinate to be one of the most common findings in patients undergoing revision surgery.^3^, ^10^, ^11^, ^12^, ^23^ Ramadan et al.^11^ reported a series of 398 patients who underwent FESS, with 52 requiring revision. Among these, the authors found adhesions, in 56% involving a lateralized middle turbinate. In contrast to this, we observed a middle turbinate lateralization in only 25% of our cases. Other, more recent investigations described similar findings with rates ranging from 6% to 11%.^8^, ^13^, ^19^ These results may reflect the more conservative surgical techniques established in the last decades.

MMA stenosis was seen in 9% of our patients. This is a lower rate than the typical results found in literature, ranging from 17% to 41%.^10^, ^11^, ^13^, ^24^ A possible explanation may be that other trials included patients suffering from CRSwNP. Relapsing nasal polyposis can cause obstruction of the MMA. An MMA stenosis may result in nasal discharge, crusts and opacified sinuses, represented by high scores in the Lund-Mackay system observed in the presented study. Chambers et al.^25^ reported subjective outcomes and endoscopic findings on 182 patients after FESS. They observed a correlation of poor symptom outcome and scarring of the MMA. Schaitkin et al.^24^ described a series of 91 patients undergoing FESS, of whom 23 had revision surgery within four years. Findings in this subset of patients included MMA stenosis in 17%. Most patients became symptomatic within six months.

In the presented study, we observed a persistent sphenoid pathology and ostium stenosis in 7% of all patients. This is a little lower than the rates described previously in literature, ranging from 27% to 66%.^8^, ^10^, ^13^ Once more, a possible explanation may be that other trials included patients suffering from CRSwNP.

Otto and DelGaudio^23^ examined residual anatomic abnormalities in 289 frontal sinuses of 127 patients unergoing revision FESS. Similar to our study, the most common findings were inflammatory mucosal disease in 67% of revision frontal recesses, including polyps and inflammatory edema obstructing the frontal sinus. They also frequently saw retained cells or septations blocking the frontal recess: 74% of revised frontal sinuses appeared to be obstructed by retained ethmoid cells. Of these, 13% were agger nasi cells, and 53% were other anterior ethmoid cells including the ethmoid bulla, suprabullar cells, frontal bullar cells, or supraorbital ethmoid cells. They identified residual frontal cells in 8% of frontal sinuses. This is similar to our findings. In contrast to this, other studies describe higher numbers of incomplete surgical dissection with retained agger nasi cells in 49%–73% of patients needing revision FESS.^10^, ^12^, ^19^ Several investigations found a high incidence of frontal recess scarring, ranging from 25%^11^ to 67%.^10^, ^13^ In contrast to this, only 19% of our patients had frontal recess scarring. A possible reason might be the conservative FESS strategy established in the last years. Not all patients receive surgery of the frontal or the posterior ethmoid cells during the first operation. This might be an explanation for the relatively low rate of scarring in this region. Our results show a high number of variants of the frontal sinus anatomy, classified according to the International Classification of the radiological Complexity (ICC) of frontal recess and frontal sinus.^16^ The frontal sinus is considered the most challenging sinus to address surgically due to its complex anatomy and close proximity to the orbit and lateral lamella of the cribriform plate.^26^ For this reason it is important to assess the difficulty of the planned sinus surgery in this region preoperatively. A profound knowledge of the anatomical variants of the frontal sinus is indispensable.

In addition to surgical factors, the chronic and recurrent nature of the mucosal sinonasal disease is another main cause of primary FESS failure. Postoperative medical therapy is essential for disease control.^12^ However, inadequate primary surgery may not only cause persistence of symptoms, but also impedes sufficient access of topical pharmacotherapy to the paranasal sinuses. The weight of adequate resection of obstructing structures is therefore intensified.

## Conclusion

The weight of an accurate surgical technique during primary FESS is underscored by reports of worse outcome after revision surgery. In addition, revision FESS has increased complication rates. Several studies report inadequate resection of obstructing structures to be a principal risk factor for recurrent CRS and the need for revision FESS. In the presented study we analyzed the data of a large patient collective presenting with CRSsNP and requiring revision FESS. We identified iatrogenic causes as incomplete ethmoidectomy, a residual uncinated process, frontal recess scarring, middle turbinate lateralization and MMA stenosis to be the most frequent findings in patients undergoing revision FESS. The risk of scarring in the case of circumferential mucosal injury and the creation or inadequate resection of obstructing structures seems to be an important etiologic factor for primary FESS failure. As with many other conditions, it is likely that the first chance to cure is the best chance. Meticulous attention in the area of the ostiomeatal complex during surgery with ventilation of obstructed anatomy as well as avoidance of scarring and turbinate destabilization may reduce the failure rate after primary FESS. This should be considered during the surgical training.

## Conflicts of interest

The authors declare no conflicts of interest.

## Funding

This research did not receive any specific grant from funding agencies in the public, commercial, or not-for-profit sectors.
